# Lysine Succinylation Contributes to Aflatoxin Production and Pathogenicity in Aspergillus flavus*[Fn FN2]

**DOI:** 10.1074/mcp.RA117.000393

**Published:** 2018-01-03

**Authors:** Silin Ren, Mingkun Yang, Yuewei Yue, Feng Ge, Yu Li, Xiaodong Guo, Jia Zhang, Feng Zhang, Xinyi Nie, Shihua Wang

**Affiliations:** From the ‡Key Laboratory of Pathogenic Fungi and Mycotoxins of Fujian Province, and School of Life Sciences, Fujian Agriculture and Forestry University, Fuzhou, 350002, China;; §Key Laboratory of Algal Biology, Institute of Hydrobiology, Chinese Academy of Sciences, Wuhan 430072, China

## Abstract

*Aspergillus flavus* (*A. flavus*) is a ubiquitous saprophytic and pathogenic fungus that produces the aflatoxin carcinogen, and *A. flavus* can have tremendous economic and health impacts worldwide. Increasing evidence demonstrates that lysine succinylation plays an important regulatory role in metabolic processes in both bacterial and human cells. However, little is known about the extent and function of lysine succinylation in *A. flavus*. Here, we performed a global succinylome analysis of *A. flavus* using high accuracy nano-LC-MS/MS in combination with the enrichment of succinylated peptides from digested cell lysates and subsequent peptide identification. In total, 985 succinylation sites on 349 succinylated proteins were identified in this pathogen. Bioinformatics analysis revealed that the succinylated proteins were involved in various biological processes and were particularly enriched in the aflatoxin biosynthesis process. Site-specific mutagenesis and biochemical studies showed that lysine succinylation on the norsolorinic acid reductase NorA (AflE), a key enzyme in aflatoxins biosynthesis, can affect the production of sclerotia and aflatoxins biosynthesis in *A. flavus.* Together, our findings reveal widespread roles for lysine succinylation in regulating metabolism and aflatoxins biosynthesis in *A. flavus*. Our data provide a rich resource for functional analyses of lysine succinylation and facilitate the dissection of metabolic networks in this pathogen.

It is well accepted that post-translational modifications (PTMs)^1^ of proteins are essential mechanisms for diversifying protein functions and controlling many biological processes (1, 2). Among the hundreds of different PTMs, lysine succinylation was recently identified by Zhang (3), and succinyl-CoA was presumed as a cofactor (3). Succinylation has been identified in both histone and non-histone proteins, and in both prokaryotes and eukaryotes (4, 5). It has been reported that succinylation is involved in the regulation of many cellular functions (5, 6). Accumulating evidence suggests that this novel modification can overlap with acetylation and malonylation to regulate metabolic pathways and other developmental processes (4, 5, 7, 8). Moreover, many metabolic enzymes and antibiotic resistance proteins in *Mycobacterium tuberculosis* have been identified with succinylation, providing a basis for further exploration of the pathophysiological role of succinylation (9). Succinylation is also reported to play roles in adaptations to changes in carbon sources (8, 10). However, to our knowledge, no study of lysine succinylation in *Aspergillus flavus* (*A. flavus*) has yet been reported.

*A. flavus* is a common and ubiquitous fungus that can threaten to human life and the living of it. It is primary causative agent of chronic indolent invasive sinonasal infection and the second main cause of aspergillosis in human (11). In addition, *A. flavus* produces a kind of toxic, carcinogenic, teratogenic and mutagenic secondary metabolite, aflatoxin, which can cause acute death, liver cancer, and chronic toxicity in both animals and human (12). Many crops can also be affected by *A. flavus* and aflatoxins (13, 14). To understand the detrimental impacts of *A. flavus* and the aflatoxins, it is important to explore the details of aflatoxin biosynthesis, metabolic process and the growth rhythm of *A. flavus*. In our previous studies, environmental factors, including temperature and water activity, were considered the main factors that control aflatoxin production (15, 16). In addition, carbon source is another factor to regulate aflatoxin biosynthesis, as demonstrated by previous studies (17–22). It has been proved that lysine succinylation plays an important role in the response to carbon source shift and plays a pathophysiological role in the pathogen (8, 9, 23). In our preliminary experiments with *A. flavus*, the succinylation and aflatoxin production levels were changed dramatically in response to different carbon sources in synthetic media. We speculated that lysine succinylation may be one mechanism in the regulation of aflatoxin production in *A. flavus*. However, no succinylated proteins are known in *A. flavus*, which is an impediment for understanding the functions of lysine succinylation in this pathogen.

To fill this knowledge gap, a systematic study of the functions of lysine succinylation in *A. flavus* was carried out. First, a global analysis of the lysine succinylome in *A. flavus* with high resolution mass spectrometry was performed, and 985 succinylation sites in 349 succinylation proteins were identified. Bioinformatics analysis of succinylation proteins and succinylation sites revealed that succinylation was involved in diverse cellular and metabolic processes. Furthermore, the succinylation modifications were also identified in proteins that belonged to the aflatoxin biosynthesis pathway. We speculated that succinylation may play an important role in the regulation of aflatoxins production. To test this hypothesis, the functional significance of lysine succinylation sites on the norsolorinic acid reductase NorA (AflE), a key enzyme in aflatoxins biosynthesis, was confirmed by site-specific mutagenesis and biochemical studies. The results demonstrated that succinylation of AflE can decrease the production of sclerotia and aflatoxin B_1_. We provided the first evidence that succinylation may be a mechanism involved in aflatoxin biosynthesis in *A. flavus*.

## EXPERIMENTAL PROCEDURES

### 

#### 

##### Strains, Media, and Cultivation Conditions

*A. flavus* NRRL 3357 was obtained from Prof. Zhumei He (Sun Yat-Sen University, Guangzhou, China). The *A. fumigatus* Af293 and *A. flavus CA14 PTs* strain were provided by Dr. Yang Liu (Institute of Food science and Technology, Chinese Academy of Agricultural Sciences, Beijing, China) and Chang P. K. (Southern Regional Research Center, Agricultural Research Service, U. S. Department of Agriculture, United States) (24). *A. flavus* was cultured in liquid YES medium (20 g/L yeast extracts; 150 g/L sucrose; 1 g/L MgSO_4_●7H_2_O) (25) or solid YES medium with agar (15 g/L) (26). Conidia obtained from agar slant cultures were cultured on a rotary shaker in the dark for 7 d at 28 °C (10^6^ conidia/ml liquid medium) (26, 27).

For carbon sources utilization studies, *A. flavus* was cultured in the improved media, in which sucrose was replaced with 150 g/L, 75 g/L, 37.5 g/L, 18.75 g/L sodium succinate, or 150 g/L, 37.5 g/L sodium acetate, or in which sucrose was decreased to 75 g/L (8, 28–30). For high salt stress, *A. flavus* was cultured in the improved media, in which sodium chloride was extra added to YES medium to final concentrations of 0.5 and 1 m. For colony morphology and spore formation analysis, 10^4^ spores of *A. flavus* were inoculated onto YES plates and the improved media, and then cultured at 37 °C for 4 d in the dark. Quantitative analysis of conidial production was performed as previously reported (31). For the aflatoxin production analysis, 10^6^ conidia of *A. flavus* were inoculated into liquid YES media and the improved media, and then cultured at 28 °C for 6 d in a rotary shaker (180 rpm) in the dark (26, 27). Cultures were harvested, lysed and immunoblotted with antisuccinyl lysine polyclonal rabbit antibodies (PTM Biolabs Inc., Chicago, IL).

##### Protein Preparation and In-solution Trypsin Digestion

Conidia (10^6^/ml) were inoculated with liquid YES media and the improved media, and cultured for 6 d at 28 °C. Subsequently, phenylmethanesulfonyl fluoride (PMSF, Beyotime, Jiangsu, China) was added into the cultures to inhibit the activity of endogenous proteases and the cultures were agitated for another 30 min. The mycelia grown in the YES and improved media with different amounts of sodium succinate were then collected by filtration through four layers of gauze, and washed twice with ice-cold phosphate-buffered saline (PBS). After grinding the mycelia into powders, the pellets were resuspended in RIPA lysis buffer (Beyotime) containing PMSF and protease inhibitor mixture (Roche) and shaken for 1 h at 4 °C. Cellular debris was removed by centrifugation at 7,000 g for 20 min at 4 °C. The supernatant was transferred into Millipore Amicon Ultra-15 Centrifugal Filters (Sigma) to remove pigment and other small molecules by centrifugation at 4,000 g for 30 min at 4 °C. Protein concentrations were determined with a BCA Protein Assay Kit (Tiangen, Beijing, China).

The whole lysate was precipitated using 10% trifluoroacetic acid (TFA) and 1% sodium deoxycholate, and then washed twice with ice-cold acetone. The precipitated proteins (∼2 mg) were redissolved in 50 mm ammonium bicarbonate and then in-solution digested by trypsin according to previously described (23, 32). Briefly, protein extracts were subjected to disulfide reduction with 25 mm dl-dithiothreitol (37 °C, 45 min) and alkylation with 50 mm iodoacetamide (25 °C, 20 min in the dark). Then, the sample was digested with sequencing grade modified trypsin (1:100, w/w, cleaving peptide chains mainly at the carboxyl side of the lysine or arginine amino acids) at 37 °C for 4 h, and then further digested with additional trypsin (1:100, w/w) at 37 °C for 20 h. The digestion was quenched by adding 0.1% TFA. The solution was clarified by centrifugation at 3000 × *g*.

##### Immunoaffinity Enrichment and LC-MS/MS Analysis

Succinylated peptides were enriched using agarose-conjugated antisuccinyllysine antibody (PTM Biolabs Inc.) as previously described (23). Briefly, peptides were mixed with preconjugated antisuccinyllysine antibody resin (PTM Biolabs), and incubated for 6 h at 4 °C with gentle rotation. The immunoprecipitates were washed with NETN buffer (0.5% Nonidet P-40, 1 mm EDTA, 100 mm NaCl, 50 mm Tris-HCl, pH 8.0) 3 times, with ETN buffer (1 mm EDTA, 100 mm NaCl, 50 mm Tris-Cl, pH 8.0) twice, followed by wash with water twice. The bound peptides were eluted by washing three times with 1% TFA (v/v), and desalted with self-packed C_18_ columns (40 μm, 60 Å pore size, AgilentTechnologies, Santa Clara, CA). Finally, the immune-enriched succinylated peptides were collected and dried in a SpeedVac.

The enriched peptides were dissolved in the HPLC buffer A (0.1% (v/v) formic acid in water), and analyzed by online nanoflow LC-MS/MS using an easy nLC-1000 system (Thermo Scientific) connected to a Q-Exactive (Thermo Scientific) mass spectrometer as previously described (33). Briefly, the samples were loaded onto the analytical C_18_-nanocapillary LC column (5 μm particle size, 100 Å pore diameter) and eluted with a linear gradient from 5% solvent B (90% acetonitrile/0.1% formic acid, v/v) to 80% solvent B for 40 min at a flow rate of 300 nL/min. The samples were then ionized and sprayed into a Q-Exactive mass spectrometer by a nanospray ion source. Mass spectrometer analysis was carried out in a data-dependent mode with full scans (350 to 1600 *m*/*z*) acquired using an Orbitrap mass analyzer at a mass resolution of 70,000 at *m*/*z* = 200. Fifteen most intense precursor ions from a survey scan were picked for MS/MS fragmentation by higher energy C-trap dissociation (HCD) with normalized collision energy of 27% and detected at a mass resolution of 17,500 in the Orbitrap. The automatic gain control for full FT MS was set to 3 × 10^6^ ions and for FT MS/MS was set to 5 × 10^4^ ions with a maximum injection times of 50 ms and 200 ms, respectively.

##### Data Analysis

All MS/MS data obtained from LC-MS/MS were searched against the *A. flavus* protein database downloaded from NCBI (http://www.ncbi.nlm.nih.gov/; 13,485 protein sequences, released 2013) concatenated with a reverse decoy database and common contaminants using the MaxQuant software (version 1.3.0.5) (34). The precursor and fragment ion mass tolerances were set as 10 ppm and 0.02 Da, respectively. Two missed cleavages were allowed for trypsin and fixed modification was Carbamidomethylation (Cysteine). Variable modifications were set as oxidation (Methionine), deamidation (Asparagine/Glutamine), succinylation (Lysine) and acetylayion (Protein N-terminal). Minimum peptide length was set at 6, whereas the estimated false discovery rate (FDR) thresholds for modification site, peptide and protein were fixed at 1%. All MS/MS spectra of the identified succinylation peptides were manually inspected using previously reported criteria (35). Furthermore, to improve the reliability of the MS result, all succinylation sites identified at the C-terminal were removed before the bioinformatics analysis. All raw data have been uploaded to the publicly accessible database PeptideAtlas (data set ID PASS00795) (http://www.peptideatlas.org/PASS/PASS00795) (36, 37).

##### Bioinformatics Analysis

The identified proteins were classified into biological process, molecular function and cellular component based on Gene Ontology annotation by the Blast2GO software (38). The YLoc was used to predict the subcellular localization of the succinylated proteins (39, 40). Functional enrichment analysis were performed by DAVID for the GO terms, KEGG (Kyoto Encyclopedia of Genes and Genomes) pathways and Pfam domains (41) and the corresponding *p* value < 0.05 (Hypergeometric test) was considered statistically significant. Specific motifs were analyzed using the Motif-X website (42, 43) with a significant 0.000001 and an in-house script as previously reported (44). The NetSurfP website was used to predict the secondary structures of all succinylated proteins (45). Protein-protein interactions of the succinylation proteins were generated by STRING (46, 47) and visualized using the Cytoscape software (version 2.8.3) (48). The homology analysis was performed by ClustalW using the protein sequences from the NCBI website, and the Neighbor-Joining (NJ) phylogenetic tree was constructed using MEGA6.0 (49).

##### Western Blot Analysis

The protein extracts (50 μg) were separated by 12% SDS/PAGE and transferred to a polyvinylidene difluoride (PVDF) membrane (GE Healthcare, Piscataway, NJ). The membrane was blocked at ambient temperature for 2 h in TBST (25 mm Tris·HCl, pH 8.0, 125 mm NaCl, 0.1% Tween 20) containing 5% (w/v) BSA. Then, the membrane was incubated with the succinyllysine polyclonal rabbit antibody (PTM Biolabs, Inc.) at a 1:2000 dilution overnight at 4 °C. After washing with TBST four times for 15 min each, the membrane was incubated with horseradish peroxidase-conjugated antirabbit IgG (1:2000 dilutions, ABGENT) at ambient temperature for 1 h. The G:BOX Chemi XT4 system (SYNGENE) was used for signal detection.

##### Generation of aflE *Mutant and Point Mutants*

The *A. flavus* gene deletion mutant and point mutants were constructed and confirmed using previously described methods (49–52). Briefly, we used *A. fumigatus* gene *pyrG* as a selectable nutritional marker. The upstream (1411 bp) and downstream fragment sequences (1443 bp) of *aflE* in *A. flavus*, as well as the full-length *A. fumigatus* Af293 gene *pyrG* (1891 bp) were amplified by PCR with the primers provided in supplemental Table S1 and three of the fragments were then fused into the interruption fragment (^up^*aflE-pyrG*-^down^*aflE*, 4048 bp) using the fusion PCR approach. Finally, the fusion PCR product was transformed into protoplasts (*A. flavus CA14 PTs* strain) as the *aflE* deletion strain (Δ*aflE*), whereas the *pyrG* gene was also transformed into protoplasts as the wild type (WT) strain using the modified polyethylene glycol method (53). The *aflE* gene was inactivated by the homologous recombination strategy and the transformants of Δ*aflE* and the WT strains were selected on YES media without uracil and by PCR until the mutants contained interrupted genes. For construction of the complementary strain (Δ*aflE::aflE*), the *aflE* gene containing the upstream promoter element and 3′-non-translated region was amplified from *A. flavus* by PCR with primers HindIII-*aflE* and SmaI-*aflE* (*aflE*-C-F and *aflE*-C-R in supplemental Table S1). The amplification product was inserted into the Chromosomal Integrating Shuttle Vector pPTR I (TAKARA, Japan) containing spyrithiamine resistance gene (*ptrA*). Finally, the complementary plasmid (pPTR-*aflE*) was transformed into the Δ*aflE* mutant and selected on YES media containing pyrithiamine. For the generation of site-directed mutants, the complementary plasmid was used as the template, and point mutation vectors were induced by PCR with mutagenic primers (supplemental Table **S**1) containing the base pair substitution as previously described (54, 55). Transformations were performed in the Δ*aflE* strain and the transformants were selected on YES media containing pyrithiamine. The succinylated residue of AflE at K370 was replaced by alanine and arginine residues to prevent succinylation in the K370A and K370R point mutated strains. The point mutations in the transformed cells were further confirmed by DNA sequencing analysis.

##### Southern Blot Analysis and Quantitative Real-Time PCR

The WT, Δ*aflE*, Δ*aflE::aflE*, K370A, and K370R strains were further verified by Southern blot with the North2South™ Biotin Random Prime DNA Labeling Kit (No. 17075, Thermo Scientific) and North2South™ Chemiluminescent Hybridization and Detection Kit (No. 17097, Thermo Scientific), according to a previous study (56). Simply, genomic DNA from each strain was singly digested with *EcoR* I and hybridized with a 0.959 kb probe of the upstream region fragment of *aflE*. For quantitative real-time PCR, mycelia were harvested after incubation in YES media at 28 °C for 48 h in the dark, and immediately ground in liquid nitrogen. RNA was isolated from 100 mg of ground mycelia with the Eastep Total RNA Extraction Kit (Promega) and purified with RNase-free DNase I (Thermo Scientific). cDNA was synthesized with the RevertAid First Strand cDNA Synthesis Kit (Thermo Scientific). Subsequently, qPCR was performed on a PikoReal Real-Time PCR machine (Thermo Scientific) using the SYBR Green qPCR mix (TAKARA) (primers listed in supplemental Table S1). The expression of the *aflE* gene was analyzed and the *actin* gene was used as an endogenous control. The REST 2009 software was used to calculate the relative expression of target genes with the PairWise Fixed Reallocation Randomization Test (57).

##### Phenotypic Analysis

To analyze growth and conidia formation, 1 μl of a 10^6^ spores/ml suspension of *A. flavus* conidia was point inoculated onto solid YES media and PDA media according to previously reported (49, 58). Cultures on YES media were grown for 4 d at 37 °C in dark and the colony morphology was recorded. Conidia cultured on PDA were cultured at 37 °C in the dark, then collected with 7% DMSO and 0.5% Tween-20 and counted using a hemocytometer. Cultures on YES media were grown for 2 d and used to observe conidiophores. For the sclerotial production analysis, 10^4^ conidia were inoculated onto Wickerham (WKM) medium (59). Cultures were grown for 10 d at 37 °C in dark and sclerotial formation was recorded for the WT and mutant strains. The plates were then sprayed with 75% ethanol to kill and wash away conidia to aid in enumeration of the sclerotia. Each experiment was performed with four replicates for three times.

For the analysis of aflatoxin production, 10^6^ *A. flavus* conidia were incubated in YES liquid media with shaking at 180 rpm in the dark at 28 °C for 6 d. Aflatoxins were extracted with an equal volume of chloroform with agitation for 30 min. Aflatoxins dissolved in the chloroform were collected via centrifugation and separated using a separatory funnel. After drying at 70 °C, thin layer chromatography (TLC) was used to analyze aflatoxins biosynthesis. 5 μl solution was loaded onto a silica gel plate (200 × 200 mm, GF254, Qingdao Haiyang Chemical Co., Ltd., Qingdao, China), and separated using acetone/chloroform (10:90, v/v). Then, silica gel plates were exposed to UV radiation and captured at 312 nm wavelength, and the aflatoxin B_1_ content of the samples was analyzed with a JD-801 Computer-aided Image Analysis System (JEDA Co., Nanjing, China) by visual comparison to standard aflatoxin B_1_ (Sigma, USA). For further analysis of aflatoxins production, HPLC was also used to confirm the presence of aflatoxins in the samples as previously described (49). The samples were analyzed by HPLC (Breeze HPLC, Waters) using a MYCOTOX C_18_ column (NO. 1612124, 250 × 4.6 mm, Pickering Laboratories) at 42 °C. After equilibration with the running solvent (water-methanol-acetonitrile, 56:22:22), a total of 20 μl aliquot from the chloroform layer was injected and run for 15 min (1.0 ml/min). Aflatoxins were detected with a fluorescent detector. The emission wavelength was 455 nm and the excitation wavelength was 365 nm.

##### Host Infection Assay

The ability of the WT and mutant strains to infect peanuts was measured as described previously (31, 60). The peanut cotyledons were rinsed three times with 0.05% sodium hypochlorite, 75% ethanol and sterile water. Sterilized seeds were then transferred into 250 ml sterilized flasks and inoculated with 10^5^ spores/ml suspension for 30 min with continuous shaking at 50 rpm. A blank control was performed by inoculating the peanut cotyledons with sterile water. The peanut cotyledons were placed in culture dishes lined with three pieces of moist sterile filter paper to maintain humidity. After 4 d incubation at 28 °C in the dark, the infected peanut cotyledons were harvested in 50 ml tubes, and then vibrated for 2 min to release the spores in 15 ml of 0.05% Tween 80 (v/v in water). Conidia were counted hemocytometrically. Furthermore, an equal amount of chloroform was added to extract aflatoxins as previously described (52). Each treatment of peanuts was performed with four replicates for three times.

##### Safety Procedures

*A. flavus* and *A. fumigatus* Af293 were cultivated in biosafety cabinets with the protection of latex gloves and respirator masks. The biological garbage was autoclaved at 121 °C for 30 min and then treated with sodium hypochlorite.

##### Experimental Design and Statistical Rationale

The succinylome analysis of *A. flavus* was tested by analyzing a total of five different biological models subjected to different carbon source or different concentration of sodium succinate, because of changes of the succinylome in response to carbon source (8, 10). Then, succinylated peptides were enriched using immunoaffinity enrichment strategies and analyzed by high-accuracy nanoflow LC-MS/MS. FDR threshold parameters of database searching for every protein, peptide, and modification site were set to maximum 1%. Database of the proteome of *A. flavus* was downloaded from NCBI (http://www.ncbi.nlm.nih.gov/; 13,485 protein sequences, released 2013). GO enrichment was performed by DAVID (41) using Hypergeometric test and the corresponding *p* value < 0.05 (Hypergeometric test) was considered statistically significant. Motifs of succinylated peptides were analyzed by Motif-X (42, 43) with a significance level of 0.000001. Secondary structures were predicted using NetSurfP (45), with *p* values calculated by Wilcoxon test.

In the functional study, three replicates were prepared and analyzed for each of the biological samples: WT, Δ*aflE* strain, Δ*aflE:aflE* strain and site-directed mutants (K370R and K370A), which represented the non-succinylated lysine of AflE. All mutants were constructed and verified by Southern blot analysis and Western blot analysis. The following analyses were performed on WT, Δ*aflE* strain, Δ*aflE:aflE* strain, K370R and K370A: growth, conidiophores, conidia, sclerotia, aflatoxin production, and seed infection. The means and standard deviations of three independent experiments were provided and the statistical tests used to analyze the data are indicated by one-way ANOVA.

## RESULTS

### 

#### 

##### Identification of Lysine Succinylation in A. flavus

**Fig. 1.**
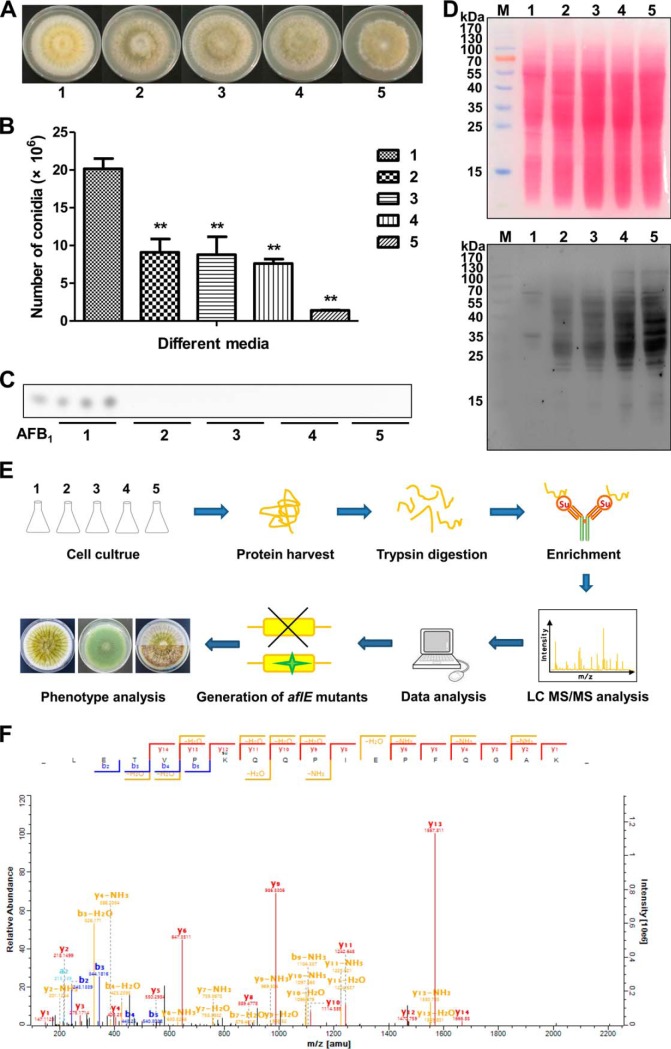
**Profiling lysine propionylation in *A. flavus* NRRL 3357.** *A*, Morphological phenotypes of *A. flavus* on different media. 1: standard YES media, 2: improved media, in which sucrose was replaced by 18.75 g/L sodium succinate, 3: improved media, in which sucrose was replaced by 37.5 g/L sodium succinate, 4: improved media, in which sucrose was replaced by 75 g/L sodium succinate, 5: improved media, in which sucrose was replaced by 150 g/L sodium succinate. *B*, Quantitative analysis of spore grown on different media (*p* value < 0.01). *C*, Thin-layer chromatography analysis of aflatoxin production of *A. flavus* grown on different media. *D*, Ponceau staining of protein lysates from *A. flavus* grown on different media and Western blotting analysis of lysine succinylation in *A. flavus* grown on different media. *E*, Workflow for lysine succinylome analysis of *A. flavus. F*, A representative MS/MS spectrum of a succinylated peptide from the norsolorinic acid reductase NorA (AflE).

We previously performed a succinylome analysis of the pathogenic *Mycobacterium tuberculosis*, indicating that the significant change of lysine succinylation level resulted from various carbon sources in media (23). To this end, we performed Western blot assay to investigate whether the relative abundance of lysine succinylation level would change under different carbon sources in *A. flavus*. The pathogenic fungus *A. flavus* was grown in the improved media containing different concentrations of sucrose, sodium succinate, sodium acetate and sodium chloride. As expected, we obtained that the hyphal extension rate of *A. flavus* was significantly slower when the strains were grown in these improved media, suggesting an inhibitory effect of these different carbon sources (sodium succinate, sodium acetate and sucrose) and sodium chloride on the growth of *A. flavus* (Fig. 1*A* and supplemental Fig. S1*A*, S2*A*, S3*A*). Accordingly, the conidia color of *A. flavus* turned green in the presence of sodium succinate (Fig. 1*A*), whereas the conidia color of *A. flavus* turned white when the strains were grown in the acetate-containing media (supplemental Fig. S1*A*). However, the different concentrations of sucrose and sodium chloride did not cause the change of the conidia color of *A. flavus* (supplemental Fig. S2*A* and supplemental Fig. S3*A*). Our observations revealed that these different carbon sources (sodium succinate, sodium acetate and sucrose) and sodium chloride could additionally lead to significant alterations in the morphology and cell surface properties. Furthermore, a significant decrease in conidia production occurred in *A. flavus* under these improved media when compared with *A. flavus* grown in YES media (Fig. 1*B* and supplemental Fig. S1*B*, S2*B*, S3*B*). Because the growth and aflatoxin biosynthesis could be affected by different carbon sources in *Aspergillus* (19, 22, 61–63), we next sought to assess whether aflatoxin biosynthesis would be affected by the improved media using TLC analysis. As shown in Fig. 1*C* and supplemental Fig. S1*C*, *A. flavus* failed to produce aflatoxin in response to succinate and sodium acetate. Also, we found that aflatoxin production was not inhibited when *A. flavus* were grown in the improved media containing sucrose (supplemental Fig. S2*C*), whereas a significant increase of aflatoxin production was obtained in *A. flavus* response to sodium chloride (supplemental Fig. S3*C*). Therefore, we anticipated that aflatoxin synthesis could possibly be induced or inhibited by a wide variety of carbon sources and salt stress. Notably, strong succinylation immunoblot signals were observed by Western blotting analysis using antisuccinyllysine antibody on the whole cell extracts, indicating the presence of lysine succinylation in *A. flavus.* More importantly, *A. flavus* exhibited higher succinylation levels under these improved media containing (sodium succinate, sodium acetate, sucrose and sodium chloride, compared with the *A. flavus* grown in YES media (Fig. 1*D* and supplemental Fig. S1*D*, S2*E*, S3*E*), suggesting that different growth media may alter the lysine succinylation profile as previously described (8). Although the mechanism remains unknown, these results imply that lysine succinylation is likely linked to carbon metabolism, growth and conidia formation in *A. flavus* and the reversible succinylation could be an important mechanism for regulating the growth and conidia formation of *A. flavus*.

To identify the lysine succinylation sites in *A. flavus*, we performed a proteomic survey of lysine succinylation by combining immunoaffinity enrichment and a MS-based high-throughput proteomic approach (Fig. 1*E*). In total, 985 high-confident succinylation sites were identified from 349 succinylated proteins with an FDR below 1% for modified peptides. The details of identified succinylation peptides are listed in supplemental Table S2. The raw data and annotated peptide spectra for all succinylated peptides have been uploaded to a public database PeptideAtlas (data set ID PASS00795). Fig. 1*F* shows an MS/MS spectrum of a succinylated peptide from norsolorinic acid reductase NorA (AflE). Moreover, the overall absolute peptide mass accuracy was 0.8183 ppm (standard deviation, 0.8829 ppm) and the average peptide score was 112.7398 (supplemental Fig. S4), further confirming the high accuracy and reliability of the modified peptide data obtained from MS.

##### Functional Annotation of Identified Succinylated Proteins

To better investigate the protein succinylation events in *A. flavus*, all succinylated proteins were assigned to the biological processes, molecular functions and cellular components categories (supplemental Fig. S5 and supplemental Table S3). The major biological processes of the 349 succinylated proteins included cellular process (32.41%), metabolic process (30.25%), cellular component organization or biogenesis (12.15%), responses to stimuli (6.71%), single-organism process (6.46%), localization (5.95%), biological regulation (2.15%), developmental process (2.03%), signaling (1.27%), multiorganism process (0.38%) and growth (0.25%) (supplemental Fig. S5*A*), indicating that succinylation may play an important role in cellular metabolism. In molecular function, the succinylated proteins were mostly involved in catalytic activity (199), structural molecule activity (65), binding (55), transporter activity (21), molecular transducer activity (4) and enzyme regulator activity (3) (supplemental Fig. S5*B*). Accordingly, most succinylated proteins were assigned to intracellular part (39.51%), followed by organelle (30.86%), further suggesting the importance of protein succinylation in metabolism. It's noteworthy that there were many modified proteins involved in macromolecular complex (13.76%), membrane (8.29%), membrane-enclosed lumen (0.18%) and extracellular region (7.41%) (supplemental Fig. S5*C*). The website YLoc was used to analyze the subcellular locations of succinylated proteins (supplemental Table S4), and 146 proteins were predicted to be located in the cytoplasm, 85 in the mitochondrion, 71 in the nucleus and 47 in the secretory pathway. Based on these results, it was verified that succinylation modification was connected to protein functionality and the metabolic network.

**Fig. 2.**
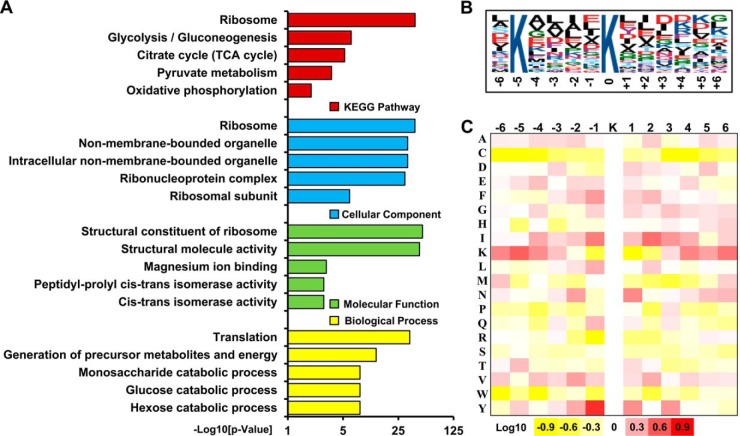
**Enrichment analysis of succinylated proteins and bioinformatics analysis of succinylation sites.** *A*, Histogram representations of the enrichment of identified succinylated proteins for biological processes, molecular functions, cellular components and KEGG pathways. The enrichment of GO categories, pathway and domain were performed using DAVID bioinformatics tools (*p* < 0.05). *B*, Motif-X analysis of the succinylated sites. The motifs with significance of *p* < 0.000001 are shown. *C*, Heat map showing sequence motifs of lysine-succinylated sites. The intensity map shows the relative abundance for ± 6 amino acids from the lysine-succinylated site. The colors in the intensity map represent the log_10_ of the ratio of frequencies within succinyl-13-mers *versus* non-succinyl-13-mers (red shows enrichment, yellow shows depletion).

We further performed an enrichment analysis using the Kyoto Encyclopedia of Genes and Genomes (KEGG) and Gene Ontology (GO) (Fig. 2 and supplemental Table S5). In the KEGG metabolic pathway analysis, the most enriched categories for succinylated proteins were ribosome (*p* = 1.64E-41) and energy metabolism, such as glycolysis/gluconeogenesis (*p* = 4.58E-07), citrate cycle (*p* = 5.84E-06), pyruvate metabolism (*p* = 2.66E-04) and oxidative phosphorylation (*p* = 1.06E-02) (Fig. 2*A*). Consistently, in the GO enrichment analysis, the succinylated proteins were also mostly enriched in protein expression metabolism with specific enrichment in translation and metabolism (Fig. 2*A*). The biological processes of succinylated proteins included translation (*p* = 1.55E-35), generation of precursor metabolites and energy (*p* = 7.56E-14), monosaccharide catabolic process (*p* = 6.50E-09) and glucose catabolic process (*p* = 6.50E-09). The major molecular functions for succinylated proteins were structural constituent of ribosome (*p* = 2.80E-51), structural molecule activity (*p* = 4.28E-47), magnesium ion binding (*p* = 8.50E-04), cis-trans isomerase activity (*p* = 1.41E-03), and peptidyl-prolyl cis-trans isomerase activity (*p* = 1.41E-03). As expected, a significant portion of the succinylated proteins were also significantly enriched in ribosome (*p* = 2.99E-41), followed by intracellular non-membrane-bounded organelle (*p* = 1.45E-33), non-membrane-bounded organelle (*p* = 1.45E-33) and ribonucleo protein complex (*p* = 5.93E-31). Taken together, these findings suggested that the succinylated proteins had a wide distribution of functions and were associated with the ribosome and protein expression/translation-associated events, as well as energy metabolism.

##### Analysis of Lysine Succinylation Sites

To identify site-specific succinylation motifs, we compared the occurrences of neighboring amino acids of the lysine succinylation sites identified in the present study with the entire set extracted from the *A. flavus* proteome. We found a significant preference for lysine at −5 position using Motif-X (Fig. 2*B*). To further assess if there was significant enrichment or depletion of specific amino acids with respect to the general amino acid composition of the entire proteome, a position-specific intensity map was generated as previously described (64). As expected, the result showed that lysine was also most commonly found at −5, whereas tyrosine was most commonly found at −1 (Fig. 2*C*). The different preferences for specific amino acid residues surrounding the lysine sites suggested unique substrate preferences in *A. flavus*.

To analyze the structural features of the succinylation sites in the identified proteins, we next predicted the structural features of the lysine succinylation sites in the identified proteins with NetSurfP algorithm (45). In accordance with a previous report (5), our results showed that succinylated lysine was found more frequently in an α-helix (46.4%), whereas the remaining 53.6% of the succinylated lysines were located in a β-sheet (13.4%) and in a coil (40.2%), respectively. The succinylated lysine was more frequently found in structured regions (supplemental Fig. S6).

##### Protein Interaction Networks of Succinylated Proteins in A. flavus

**Fig. 3.**
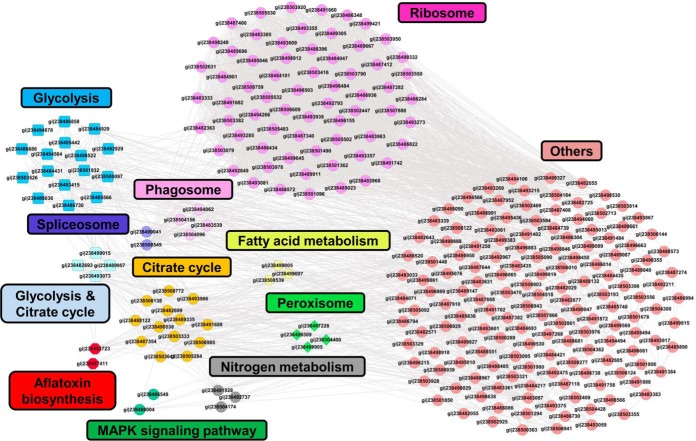
**Protein interaction networks of all identified succinylated proteins.** The interaction network was visualized with Cytoscape.

Protein-protein interaction (PPI) networks can serve as an alternative strategy to analyze physical and functional interactions. PTMs may mediate protein-protein interactions by disrupting a favorable interaction with adjacent amino acids or by providing a docking site to recruit binding partners. We generated a protein interaction network of all the identified proteins using the *A. flavus* PPI database (http://string-db.org/). The constructed protein interaction map was visualized using Cytoscape and the functional category was used to group the identified proteins (Fig. 3). A large network covering 269 succinylated proteins was constructed and many proteins involved in different pathways formed prominent and highly connected clusters. Consistent with the KEGG pathway and GO functional annotation analyses, most succinylated proteins were associated with ribosome and energy metabolism.

##### Succinylated Proteins Involved in Central Metabolism and Aflatoxins Biosynthesis

**Fig. 4.**
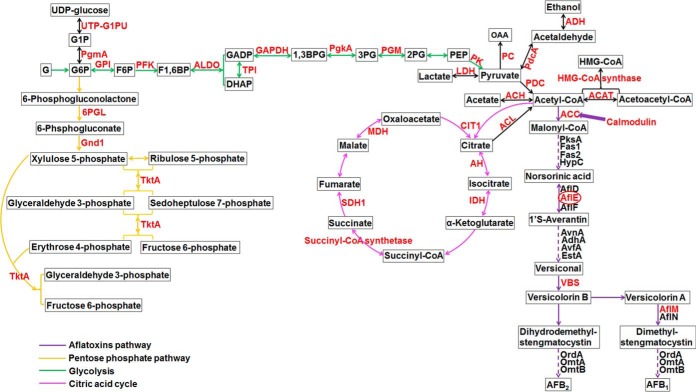
**Central metabolism and aflatoxins biosynthesis pathways in *A. flavus*.** Succinylated proteins were highlighted in red. ACC: acetyl-CoA carboxylase. PksA: noranthrone synthase, AflC/PksA/PksL1/polyketide synthase. Fas1: AflB/Fas-1/fatty acid synthase beta subunit. Fas2: AflA/Fas-2/HexA/fatty acid synthase alpha subunit. HypC: noranthrone monooxygenase, AflCa/HypC/hypothetical protein. AflD: Nor-1/norsolorinic acid ketoreductase. AflF: NorB/dehydrogenase. AvnA: averantin hydroxylase, AflG/AvnA/Ord-1/cytochrome P450 monooxygenase. AdhA: 5′-hydroxyaverantin dehydrogenase, AflH/AdhA/short chain alcohol dehydrogenase. AvfA: Oxidase, AflI/AvfA/cytochrome P450 monooxygenase. VrdA: versiconal hemiacetal acetate reductase/aryl-alcohol dehydrogenase. EstA: versiconal hemiacetal acetate esterase, AflJ/EstA/esterase. VBS: 5′-oxoaverantin cyclase/versicolorin B synthase, AflK/Vbs/VERB synthase. AflN: VerA/monooxygenase. AflM: Ver-1/dehydrogenase/ketoreductase. OmtB: DmtA, demethylsterigmatocystin 6-O-methyltransferase, AflO/OmtB/DmtA/O-methyltransferase B. OmtA: sterigmatocystin 8-O-methyltransferase, AflP/OmtA/Omt-1/O-methyltransferase A. OrdA: aflatoxin B synthase, AflQ/OrdA/Ord-1/oxidoreductase/cytochrome P450 monooxigenase. AflE: NorA/Aad/Adh-2/NOR reductase/dehydrogenase. ACAT: acetyl-CoA acetyltransferase. PDC: pyruvate dehydrogenase complex. LDH: lactate dehydrogenase. ACL: ATP citrate lyase. HMG-CoA: 3-hydroxy-3-methylglutaryl-CoA. ACH: acetyl-coA hydrolase. 6PGL: 6-Phosphogluconolactonase. 3PG: 3-phosphoglycerate. 2PG: 2-phosphoglycerate. PGM: phosphoglycerate mutase. PK: pyruvate kinase. PEP: phosphoenolpyruvate. F6P: β-d-Fructose 6-phosphate. PFK: 6-phosphofructokinase. F1,6BP: β-d-Fructose 1,6-bisphosphate. ALDO: fructose-bisphosphate aldolase. GADP: d-glyceraldehyde 3-phosphate. DHAP: dihydroxyacetone phosphate. GAPDH: glyceraldehyde phosphate dehydrogenase. 1,3BPG: d-1,3-bisphosphoglycerate. G6P: glucose-6-phosphate. G: glucose. G1P: glucose-1-phosphate. PgmA: phosphoglucomutase. PdcA: pyruvate decarboxylase. AH: aconitate hydratase. Gnd1: 6-phosphogluconate dehydrogenase. IDH: isocitrate dehydrogenase. TktA: transketolase. PC: pyruvate carboxylase. OAA: oxaloacetate. GPI: glucose-6-phosphate isomerase. MDH: malate dehydrogenase. PgkA: phosphoglycerate kinase. UTP-G1PU: UTP-glucose-1-phosphate uridylyltransferase. TPI: triose-phosphate isomerase. CIT1: citrate synthase. ADH: alcohol dehydrogenases. SDH1: succinate dehydrogenase.

We further investigated the succinylated proteins by matching them to KEGG metabolic pathways in *A. flavus* (supplemental Table S6). A large proportion of the metabolic enzymes including proteins in the pentose phosphate pathway, glycolysis and citric acid cycle, were succinylated (Fig. 4), which is consistent with the previous understanding of lysine succinylation (5, 8, 23). It's noteworthy that many proteins were involved in acetyl-CoA metabolism, which can supply precursors, acetyl-CoA and malonyl-CoA for aflatoxin biosynthesis (65–67). We anticipated that lysine succinylation may regulate the synthesis of acetyl-CoA/malonyl-CoA involved in aflatoxin biosynthesis. Most importantly, we also identified three succinylated enzymes that were directly involved in the aflatoxin biosynthesis pathway, including AflE, AflK, and AflM, which are required for conversion of acetyl-CoA to its final products, aflatoxin B_1_ and aflatoxin B_2_ (67). Our proteomic data suggested that succinylation may play an important role in metabolic pathways, especially in aflatoxin biosynthesis.

##### Effects of Succinylation on Sclerotial Development

It's widely accepted that aflatoxins, mainly produced by *A. flavus* and *A. parasiticus*, are highly toxic and carcinogenic in animals and humans. The identified succinylated proteins were likely to be involved in aflatoxin biosynthesis (*i.e.* AflE, AflK, and AflM) in our data. To test this hypothesis, we next assessed whether and how lysine succinylated protein could affect the aflatoxin biosynthesis in *A. flavus*. Based on our succinylome result, we identified a reliable succinylation site (Lys370) on a cytosolic oxidoreductase (AflE), which may play a crucial role in regulating aflatoxin biosynthesis in *A. flavus* (67). Further evolutionary conservation analysis revealed that lysine at 370 of AflE was highly conserved in the *A. flavus* orthologs, suggesting that this residue of AflE may be important for an evolutionarily conserved function (supplemental Fig. S7).

**Fig. 5.**
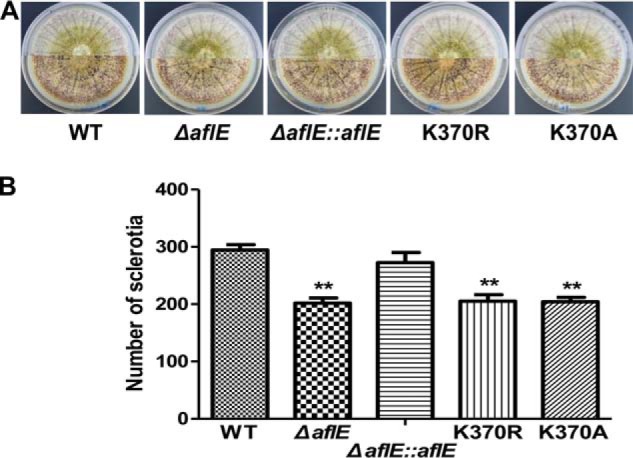
**Sclerotial characterization of different *A. flavus* strains.** *A*, Morphological phenotypes of sclerotia in WT, Δ*aflE,* K370R, K370A and Δ*aflE::aflE* strains on WKM media. *B*, Quantification analysis of sclerotia. Sclerotial production was counted from three replicates of WKM plates in (*A*). The corresponding *p* value < 0.01 was considered statistically significant.

To further gain insight into the potential function of lysine succinylation on AflE in *A. flavus*, we first made a full-length deletion mutant of the *aflE* gene using *pyrG* replacement and the site-directed mutations were generated in the *aflE* gene (supplemental Fig. S8*A* and S8*B*). The modified residue was mutated to arginine (R) or alanine (A) to prevent succinylation while maintaining the original structure (K370R, K370A) as previous studies (23, 68). The construction of the Δ*aflE* and Δ*aflE::aflE* strains was confirmed by southern blotting and RT-PCR analysis, and the site-directed mutations were further confirmed by DNA sequencing (supplemental Fig. S8*C*, S8*D*, and S8*E*). We also checked the mRNA expression and protein levels of the wild type AflE protein and its point mutations, and no significant changes were observed in any strains (supplemental Fig. S9). The sclerotial morphologies of the site-specific mutants (K370R and K370A), which represented the non-succinylated lysine of AflE, exhibited an identical sclerotial phenotypic characterization to those of the Δ*aflE* strain and those control strains (WT and Δ*aflE::aflE* strains) when grown on WKM agar medium (Fig. 5*A*). However, significant decreases in sclerotial production occurred in these mutants when compared with the WT and Δ*aflE::aflE* strains (Fig. 5*B*). Because of the significant changes in the sclerotial development of the *aflE* deletion or K370R/K370A (mimicking desuccinylation), we concluded that K370 should be desuccinylated for proper AflE-dependent regulation in sclerotial development. Moreover, we also detected the colony phenotype of each strain when grown on YES agar medium and PDA agar medium. However, the colony morphology, colony diameter, spore number and conidiophore formation of the mutants were similar to those of the WT and Δ*aflE::aflE* strains (supplemental Fig. S10). Together, it is conceivable that succinylation could play a role in regulating the sclerotial development by modulating of the AflE function.

##### Involvement of Succinylation in Aflatoxins Production in A. flavus

**Fig. 6.**
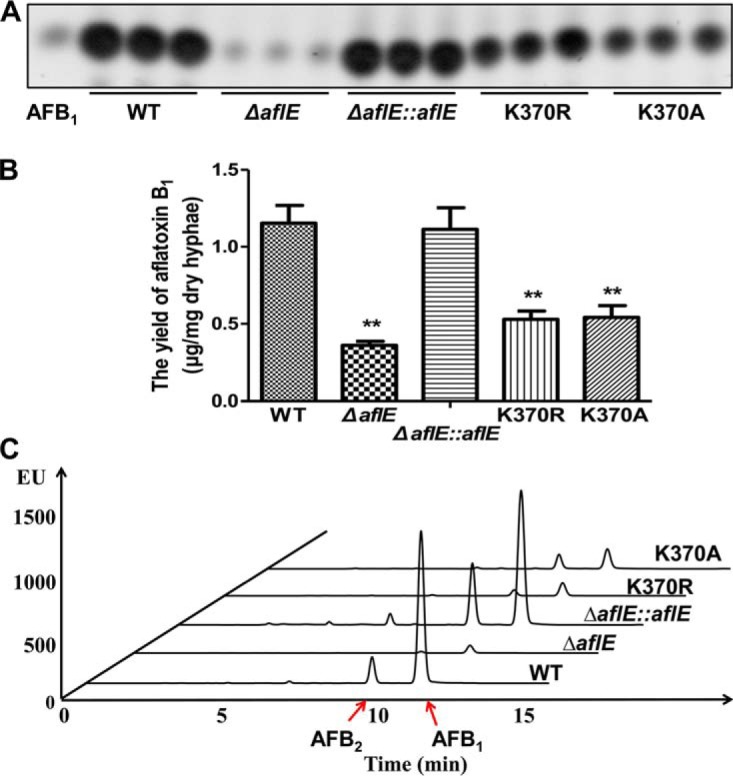
**Aflatoxin production in different *A. flavus* strains.** *A*, Thin layer chromatography analysis of aflatoxin B_1_ in WT, Δ*aflE,* K370R, K370A and Δ*aflE::aflE* strains. *B*, Quantification analysis of aflatoxin B_1_ according to the result of thin layer chromatography. The corresponding *p* value < 0.01 was considered statistically significant. *C*, HPLC analysis of aflatoxins production in WT, Δ*aflE,* K370R, K370A and Δ*aflE::aflE* strains.

*A. flavus* is well-known as a saprophytic soil fungus and the virulence of *A. flavus* is partially based on the production of aflatoxins. We further investigated if succinylation would affect aflatoxin biosynthesis using mutagenesis experiments. Notably, we detected an evident decrease in aflatoxin production in the K370R/K370A mutants compared with the WT and Δ*aflE::aflE* strains (Fig. 6*A* and 6*B*). For further analysis of aflatoxin production, HPLC was also used to confirm the presence of aflatoxins in each strain and we observed the eluted HPLC peaks corresponding to aflatoxin B_2_ (AFB_2_) and aflatoxin B_1_ (AFB_1_) in all strains. Specifically, AFB_1_ productions in WT and Δ*aflE::aflE* strains were ∼16-fold higher than that of the Δ*aflE* and K370R/K370A strains and AFB_2_ productions in the WT and Δ*aflE::aflE* strains were ∼5-fold higher than that of the K370R/K370A strains, whereas we could hardly detect AFB_2_ production in Δ*aflE* strain (Fig. 6*C*). Consequently, our results revealed that the mutation of K370 to either arginine or alanine, which mimicked the desuccinylation status of AflE, markedly affected aflatoxin biosynthesis, indicating that K370 succinylation could influence aflatoxin biosynthesis by modulating of AflE function in *A. flavus*.

##### Effects of Succinylation on Seed Infection

**Fig. 7.**
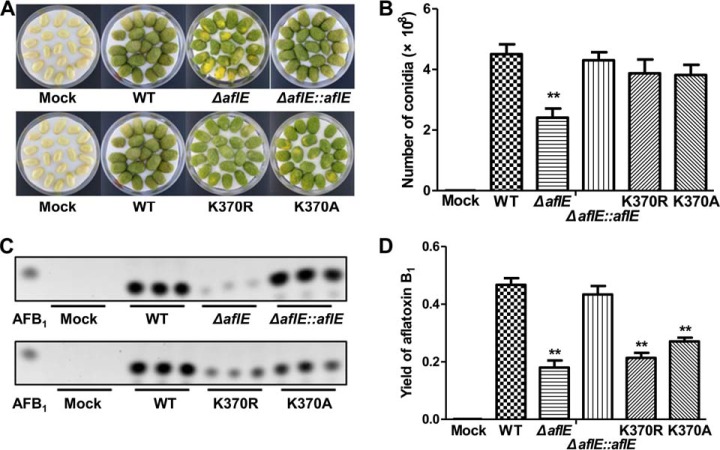
**Host colonization of different *A. flavus* strains.** *A*, Phenotypic characterization of WT, Δ*aflE,* K370R, K370A and Δ*aflE::aflE* strains on peanut cotyledons for 4 d. *B*, Quantification of conidia. Conidial production was counted from three replicates of the *A. flavus* strains in (*A*). The corresponding *p* value < 0.01 was considered statistically significant. *C*, Thin layer chromatography analysis of aflatoxin B_1_ collected from infected peanut cotyledons. *D*, Quantification of AFB_1_. AFB_1_ production was counted from three replicates of the *A. flavus* strains in (*C*). The corresponding *p* value < 0.01 was considered statistically significant.

Because *A. flavus* can be a biological hazard to crops, we inoculated all *A. flavus* strains with peanut cotyledons to further determine whether succinylation could influence infectivity and aflatoxin production after infection with *A. flavus*. In this study, we found that he Δ*aflE* strain tended to produce less conidia than the WT strain when colonizing peanut cotyledons, whereas the remaining strains appeared to grow similarly to the WT strain on peanut cotyledons (Fig. 7*A* and 7*B*). Furthermore, we measured aflatoxin production in all the strains on peanut cotyledons. Interestingly, the site-specific mutants (K370R/K370A), which represented the desuccinylation states of AflE, produced statistically less aflatoxin than the WT and Δ*aflE::aflE* strains after infecting peanut cotyledons, similar to the Δ*aflE* strain (Fig. 7*C* and 7*D*). Our results illustrated that aflatoxin biosynthesis after infection, but not the infectivity of *A. flavus* was clearly influenced by lysine succinylation.

## DISCUSSION

PTMs are chemical modifications that are involved in diversifying protein functions by regulating protein activity, stability, and cellular localization (1, 2). One such PTM is lysine succinylation, which contributes to controlling multiple biological processes, such as metabolism. In this study, with the purpose of examining the roles of succinylation in *A. flavus*, we reported the lysine succinylome in this aflatoxin-producing pathogenic fungus, which was also first reported in the filamentous fungi. Overall, 1013 unique succinylated peptides encompassing 986 high confidence succinylation sites were identified from 349 *A. flavus* proteins. The recent observations showed that nearly all metabolic enzymes major metabolic pathways are subjected to succinylation (23, 69–73), indicating the presence of a common mechanism of succinylation involving in metabolic regulation. In this study, we observed that a majority of succinylated proteins were found to be involved in ribosome, mitochondrion, glycolysis, citric acid cycle, fatty acid metabolism, ketone body synthesis, MAPK pathway, peroxisome and aflatoxins biosynthesis.

**Fig. 8.**
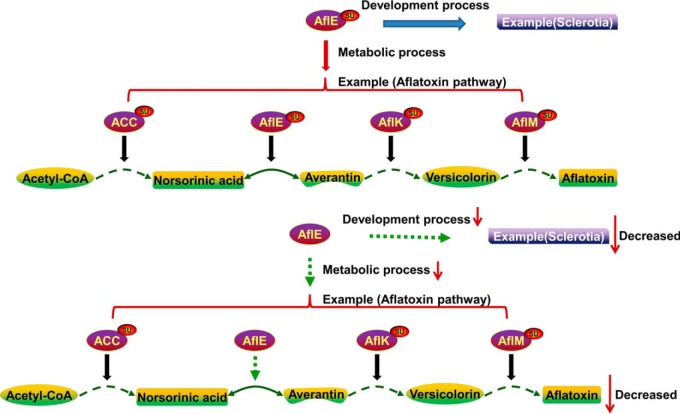
**Schematic summary of the proposed model of the role of lysine succinylation in aflatoxins biosynthesis and sclerotial formation.**

Among all metabolic pathways, glycolytic enzymes are succinylated and involved in the conversion of glucose to pyruvate in other organisms (5, 9, 23). In the present study, we found that 20 proteins within the glycolysis/gluconeogenesis pathway were succinylated, indicating a potentially conserved role of succinylation in regulating the glycolytic pathway (supplemental Table S6 and Fig. 4). In *E. coli*, the succinylated lysine residues in the TCA cycle have been shown to be important for affecting the activity of isocitrate dehydrogenase (3). In this study, we found that nearly all enzymes involved in TCA cycle were identified as targets of lysine succinylation, which is similar to the previous observation (7). Consistent with these reports, our results also suggested a potential function in the regulation of enzymatic activity by lysine succinylation in TCA cycle. Notably, the secondary metabolism, especially the aflatoxin biosynthesis pathway in *A. flavus*, is crucial for their success as pathogens. The proteins involved in aflatoxin biosynthesis were also subjected to succinylation in this work. It has been proved that AflM can convert versicolorin A to demethylsterigmatocystin or convert versicolorin B to dihydrodemethylsterigmatocystin in aflatoxin biosynthesis (67, 74, 75) and the versicolorin B synthase AflK can catalyze synthesis of versicolorin B (67). The early aflatoxin biosynthetic enzyme AflE (encoded by *norA*), a homolog of *stcV* in *Aspergillus nidulans* (76), was also identified as target of lysine succinylation (K370) in *A. flavus*. The first stable aflatoxins intermediate product norsolorinic acid (NA) can be converted to averantin by AflE (67, 77). Our data showed that the three key enzymes, including AflE, AflM and AflK, in the aflatoxin biosynthesis pathway were succinylated (Fig. 8). We hypothesized that lysine succinylation may play a crucial role in regulating the production of aflatoxins by affecting the function of these enzymatic activities. To test this possibility, we disrupted the *aflE* gene and constructed point mutant strains to investigate the potential role of lysine succinylation in development and aflatoxin biosynthesis in *A. flavus*. Our results illustrated that the number of sclerotia (Fig. 5) and aflatoxins production were evident decrease in all mutants (Fig. 6). It has already been proved that deletion of *nor-1*, the homolog of *aflE*, causes the accumulation of NA and blocks the production of aflatoxins in *A. parasiticus* (78). We speculate that the K370 is critical for AflE activity and the succinylation at K370 is the critical factor in maintaining the generation of sclerotia and aflatoxins. Likewise, although the infectivity of point mutant strains was not affected, the aflatoxin production after infection was significantly influenced (Fig. 7). Together, we propose that the K370 is critical for AflE activity and the succinylation at K370, which will induce a dramatic structural alteration at lysine residue and change the charge status of lysine residue from +1 to −1 at physiological pH (7.4) (3), is likely to affect the structure and function of AflE, subsequently resulting in the production of sclerotia and aflatoxin biosynthesis in *A. flavus*.

Furthermore, the succinylated protein acetyl-coenzyme A carboxylase (ACC) was also found to catalyze acetyl-CoA conversion to malonyl-coenzyme A, which is the initial step in fatty acids and aflatoxin biosynthesis (67, 79). In this study, six lysines in ACC were identified with succinylation. As previous study reported, calmodulin mediated activation of acetyl-CoA carboxylase, which was an important event for aflatoxin production (80). We found that the lysines at positions 22, 78, and 91 on calmodulin were succinylated, which might be important for the activity of calmodulin. In addition, other enzymes regulating the flux of acetyl-coenzyme A were also identified with succinylation. As acetyl-CoA is the substrate of aflatoxins and fatty acid, we are certain that succinylation indeed mediated aflatoxin production in *A. flavus*. Additionally, a growing body of evidence suggests that aflatoxin biosynthesis is triggered and intensified by buildup of reactive oxygen species, and peroxisomes are probably the first place for mycotoxin synthesis in fungi (81–83). In this study, three enzymes in the peroxisome, including Mn superoxide dismutase, mitochondrial peroxiredoxin Prx1 and mycelial catalase Cat1 of the antioxidant system were found to be succinylated, suggesting a possible regulation of lysine succinylation in this system. Because the aflatoxin biosynthesis and export are mediated by vesicles and endosomes (84, 85), the lysine succinylation on vesicle transport v-SNARE protein in the vesicle transport pathway might possibly affect the aflatoxin production by mediating the vesicle fusion.

In summary, we report the first large-scale, high resolution MS-based survey of lysine succinylation in the pathogenic fungus *A. flavus*. The identified succinylated proteins are involved in various biological processes and are particularly enriched in the aflatoxin biosynthesis process. Further site-specific mutations showed that lysine succinylation of AflE, a key enzyme in aflatoxin biosynthesis, could affect the formation of sclerotia and aflatoxins in *A. flavus*. Our succinylome data of *A. flavus* may help provide the accurate and detailed description of the biological roles of succinylation in this pathogenic fungus and reveals previously unappreciated roles for lysine succinylation in the potential regulation of aflatoxin biosynthesis. It will be interesting and important to determine the physiological functions affected by lysine succinylation in *A. flavus*.

## DATA AVAILABILITY

The mass spectrometry proteomics data have been deposited to the publicly accessible database PeptideAtlas (dataset ID PASS00795) (http://www.peptideatlas.org/PASS/PASS00795).

## Supplementary Material

Supplemental Data

## Footnotes

^1^ The abbreviations used are:

PTMs: post-translational modifications
PMSF: phenylmethanesulfonyl fluoride
KEGG: Kyoto Encyclopediaof Genes and Genomes
ACC: acetyl-CoA carboxylase
PksA: noranthrone synthase, AflC/PksA/PksL1/polyketide synthase
Fas1: AflB/Fas-1/fatty acid synthase beta subunit
Fas2: AflA/Fas-2/HexA/fatty acid synthase alpha subunit
HypC: noranthrone monooxygenase, AflCa/HypC/hypothetical protein
AflD: Nor-1/norsolorinic acid ketoreductase
AflF: NorB/dehydrogenase
AvnA: averantin hydroxylase, AflG/AvnA/Ord-1/cytochrome P450 monooxygenase
AdhA: 5′-hydroxyaverantin dehydrogenase, AflH/AdhA/short chain alcohol dehydrogenase
AvfA: Oxidase, AflI/AvfA/cytochrome P450 monooxygenase
VrdA: versiconal hemiacetal acetate reductase/aryl-alcohol dehydrogenase
EstA: versiconal hemiacetal acetate esterase, AflJ/EstA/esterase
VBS: 5′-oxoaverantin cyclase/versicolorin B synthase, AflK/Vbs/VERB synthase
AflN: VerA/monooxygenase
AflM: Ver-1/dehydrogenase/ketoreductase
OmtB: DmtA, demethylsterigmatocystin 6-O-methyltransferase, AflO/OmtB/DmtA/O-methyltransferase B
OmtA: sterigmatocystin 8-O-methyltransferase, AflP/OmtA/Omt-1/O-methyltransferase A
OrdA: aflatoxin B synthase, AflQ/OrdA/Ord-1/oxidoreductase/cytochrome P450 monooxigenase
AflE: NorA/Aad/Adh-2/NOR reductase/dehydrogenase
LC-MS/MS: liquid chromatography-tandem mass spectrometry
TLC: thin layer chromatography
ACN: acetonitrile
FDR: false discovery rate
GO: Gene Ontology
YES: yeast extract sucrose
FGSC: Fungal Genetics Stock Center
PBS: phosphate-buffered saline
NA: norsolorinic acid
ORF: open reading frame
TFA: trifluoroacetic acid
ACAT: acetyl-CoA acetyltransferase
PDC: pyruvate dehydrogenase complex
LDH: lactate dehydrogenase
ACL: ATP citrate lyase
HMG-CoA: 3-hydroxy-3-methylglutaryl-CoA
ACH: acetyl-coA hydrolase
6PGL: 6-Phosphogluconolactonase
3PG: 3-phosphoglycerate
2PG: 2-phosphoglycerate
PGM: phosphoglycerate mutase
PK: pyruvate kinase
PEP: phosphoenolpyruvate
F6P: β-D-Fructose 6-phosphate
PFK: 6-phosphofructokinase
F1,6BP: β-D-Fructose 1,6-bisphosphate
ALDO: fructose-bisphosphate aldolase
GADP: D-glyceraldehyde 3-phosphate
DHAP: dihydroxyacetone phosphate
GAPDH: glyceraldehyde phosphate dehydrogenase
1,3BPG: D-1,3-bisphosphoglycerate
G6P: glucose-6-phosphate
G: glucose
G1P: glucose-1-phosphate
PgmA: phosphoglucomutase
PdcA: pyruvate decarboxylase
AH: aconitate hydratase
Gnd1: 6-phosphogluconate dehydrogenase
IDH: isocitrate dehydrogenase
TktA: transketolase
PC: pyruvate carboxylase
OAA: oxaloacetate
GPI: glucose-6-phosphate isomerase
MDH: malate dehydrogenase
PgkA: phosphoglycerate kinase
UTP-G1PU: UTP-glucose-1-phosphate uridylyltransferase
TPI: triose-phosphate isomerase
CIT1: citrate synthase
ADH: alcohol dehydrogenases
SDH1: succinate dehydrogenase
NJ: Neighbor-Joining
HCD: higher energy C-trap dissociation
HPLC: high performance liquid chromatography
PPI: protein-protein interaction
WKM: Wickerham.
