# Supplementary material for: Lysine Succinylation Contributes to Aflatoxin Production and Pathogenicity in Aspergillus flavus
Source: Mol Cell Proteomics. 2018 Jan 3;17(3):457–71. doi: 10.1074/mcp.RA117.000393 (PMC5836371; doi:10.1074/mcp.RA117.000393)
Supplement: Supplemental Data [file supp_17_3_457__index.html]

Lysine Succinylation Contributes to Aflatoxin Production and Pathogenicity in Aspergillus flavus — Lysine Succinylation in Aspergillus flavus — Lysine Succinylation Contributes to Aflatoxin Production and Pathogenicity in Aspergillus flavus — Supplemental Data 

# Lysine Succinylation Contributes to Aflatoxin Production and Pathogenicity in *Aspergillus flavus*

## Supplemental Data

- All Supplemental Figures and Legends - All Supplemental Figures and Legends
- Supplementary Table 1 - Primers used in this study
- Supplementary Table 2 - Detailed informations on identified succinylated proteins and succinylated peptides in Aspergillus flavus NRRL 3357
- Supplementary Table 3 - GO classification of succinylated proteins using Blast2GO
- Supplementary Table 4 - Subcellular localization of succinylated proteins determined by Yloc
- Supplementary Table 5 - Gene-annotation enrichment of succinylated proteins using DAVID
- Supplementary Table 6 - KEGG pathways analysis of succinylated proteins
