# Supplementary material for: Lysine Succinylation Contributes to Aflatoxin Production and Pathogenicity in Aspergillus flavus
Source: Mol Cell Proteomics. 2018 Jan 3;17(3):457–71. doi: 10.1074/mcp.RA117.000393 (PMC5836371; doi:10.1074/mcp.RA117.000393)
Supplement: Supplemental Data [file supp_RA117.000393_133046_1_supp_36570_p0skbz.pdf]

Supplementary Informations

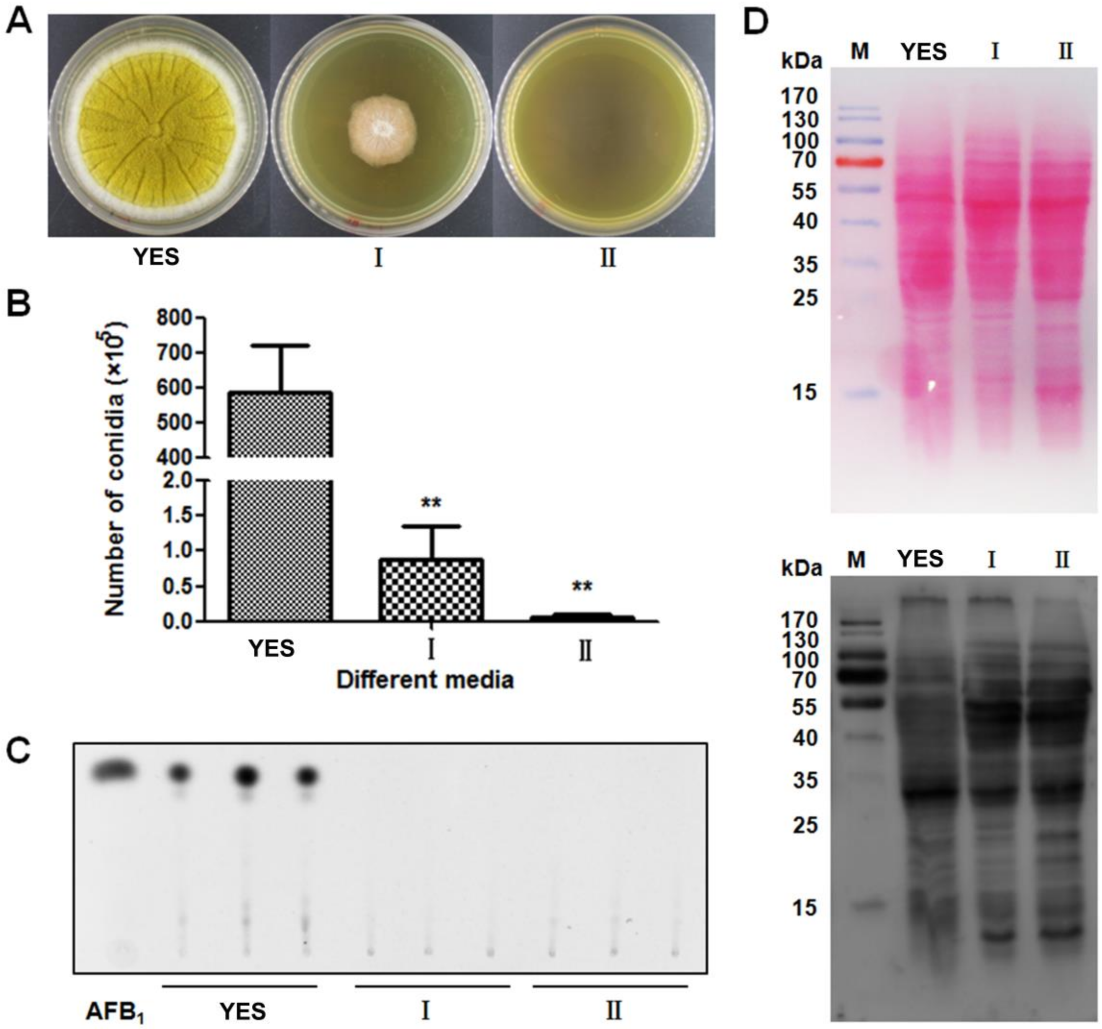

Supplementary Figure S1. Phenotypic analysis of *A. flavus* on YES medium and improved media. YES: standard YES media, I: improved media, in which sucrose was replaced with 37.5 g/L sodium acetate. II: improved media, in which sucrose was replaced with 150 g/L sodium acetate. (A) Morphological phenotypes of *A. flavus* on different media. (B) Quantitative analysis of spore grown on different media (p-value < 0.01). (C) Thin-layer chromatography analysis of aflatoxin production of *A. flavus* grown on different media. (D) Ponceau staining of protein lysates from *A. flavus* and western blotting analysis of lysine succinylation in *A. flavus* grown grown on different media.

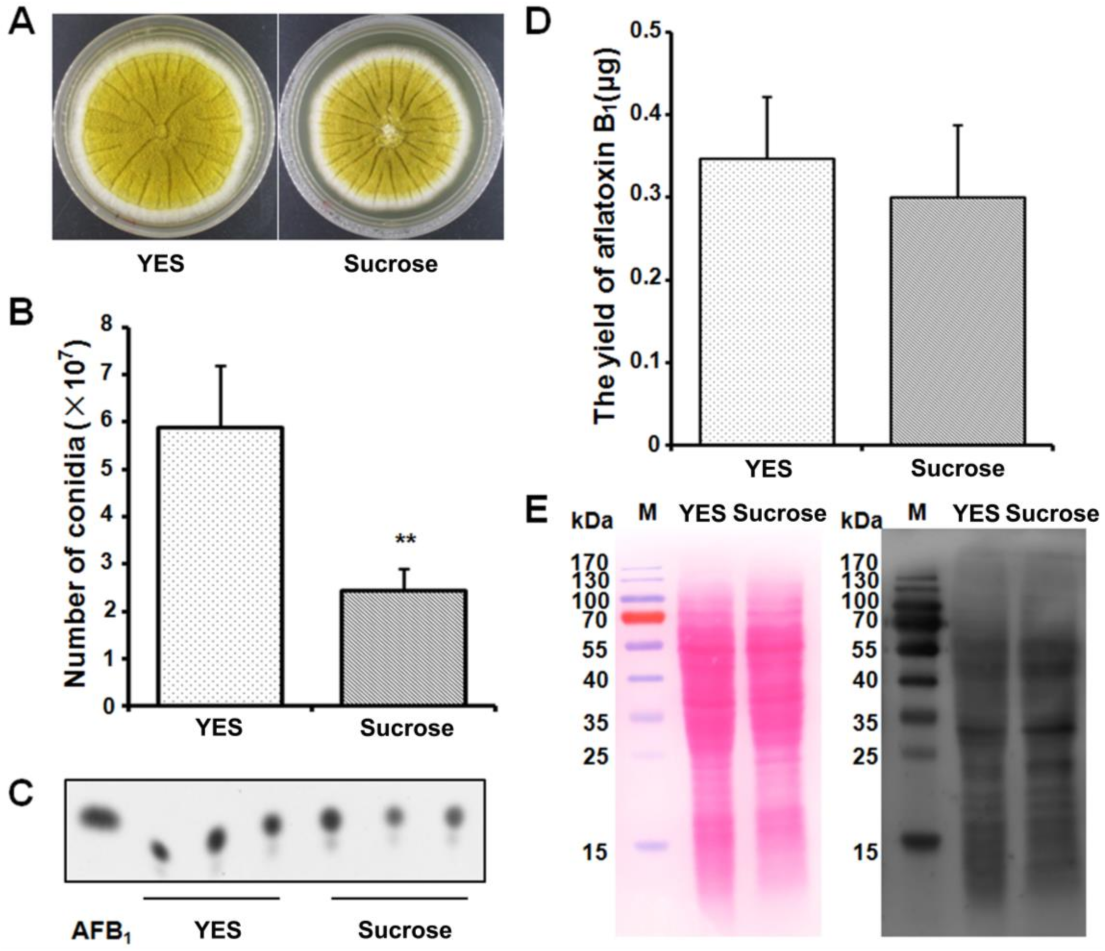

Supplementary Figure S2. Phenotypic analysis of *A. flavus* on YES medium and improved media. YES: standard YES media, Sucrose: improved media, in which sucrose was decreased to 75 g/L. (A) Morphological phenotypes of *A. flavus* on different media. (B) Quantitative analysis of spore grown on different media (p-value < 0.01). (C) Thin-layer chromatography analysis of aflatoxin production of *A. flavus* grown on different media. (D) Quantitative analysis of aflatoxin production based on the results of C. (E) Ponceau staining of protein lysates from *A. flavus* grown on different media and western blotting analysis of lysine succinylation in *A. flavus* grown on different media.

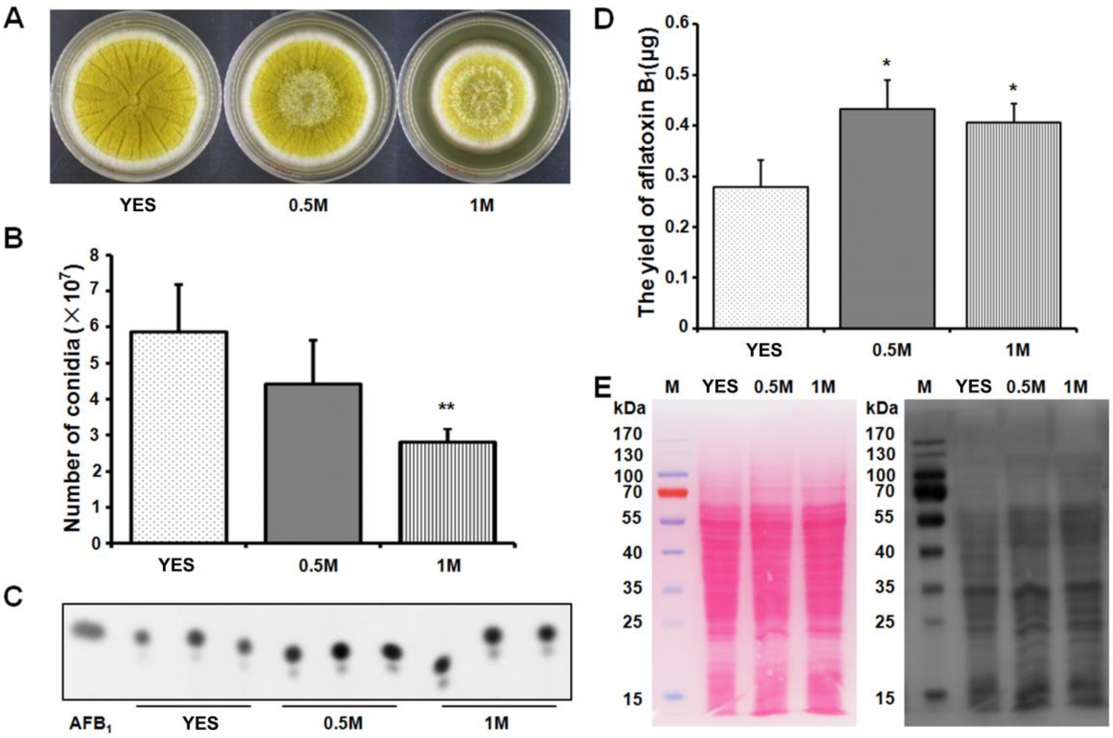

Supplementary Figure S3. Phenotypic analysis of *A. flavus* on YES medium and improved media. YES: standard YES media. 0.5M: improved media, in which sodium chloride was extra added to the final concentration to 0.5mol/L. 1M: improved media, in which sodium chloride was extra added to the final concentration to 1mol/L. (A) Morphological phenotypes of *A. flavus* on different media. (B) Quantitative analysis of spore grown on different media (p-value < 0.01). (C) Thin-layer chromatography analysis of aflatoxin production of *A. flavus* grown on different media. (D) Quantitative analysis of aflatoxin production based on the results of C (p-value < 0.05). (E) Ponceau staining of protein lysates from *A. flavus* grown on different media and western blotting analysis of lysine succinylation in *A. flavus* grown on different media.

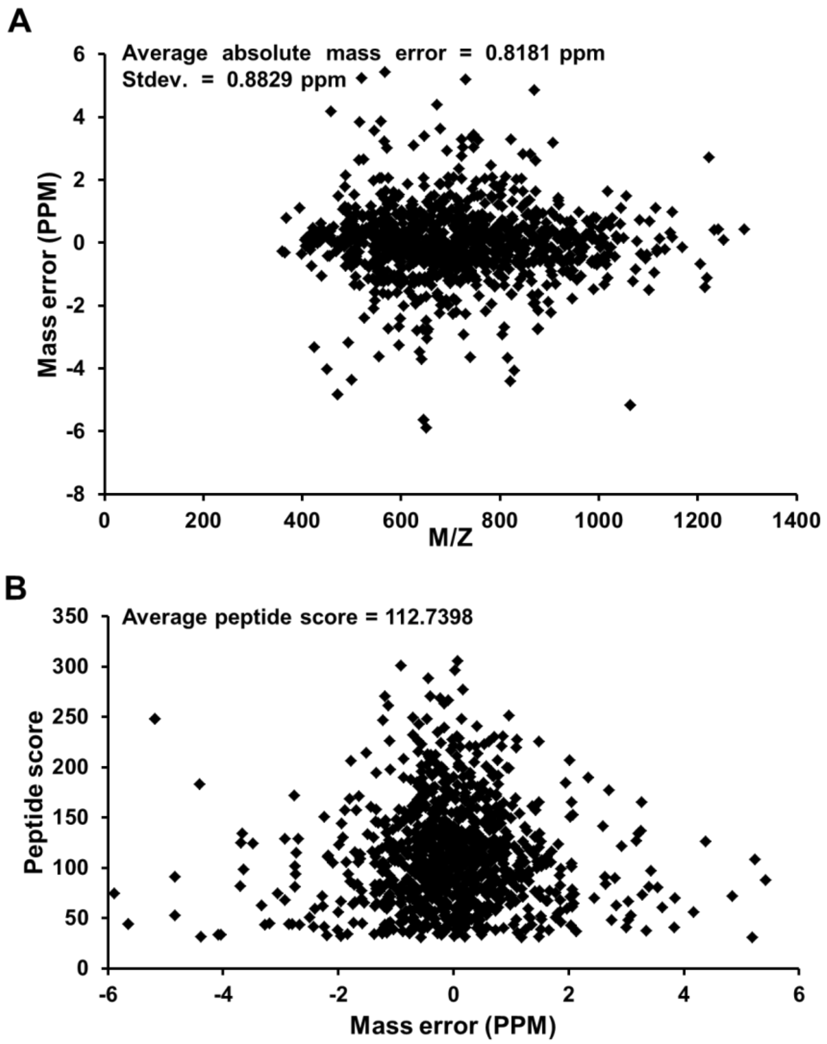

Supplementary Figure 4. Overview of the lysine succinylome in *A. flavus*. (A) Distribution of precursor mass deviations. (B) Distribution of succinylated peptide scores.

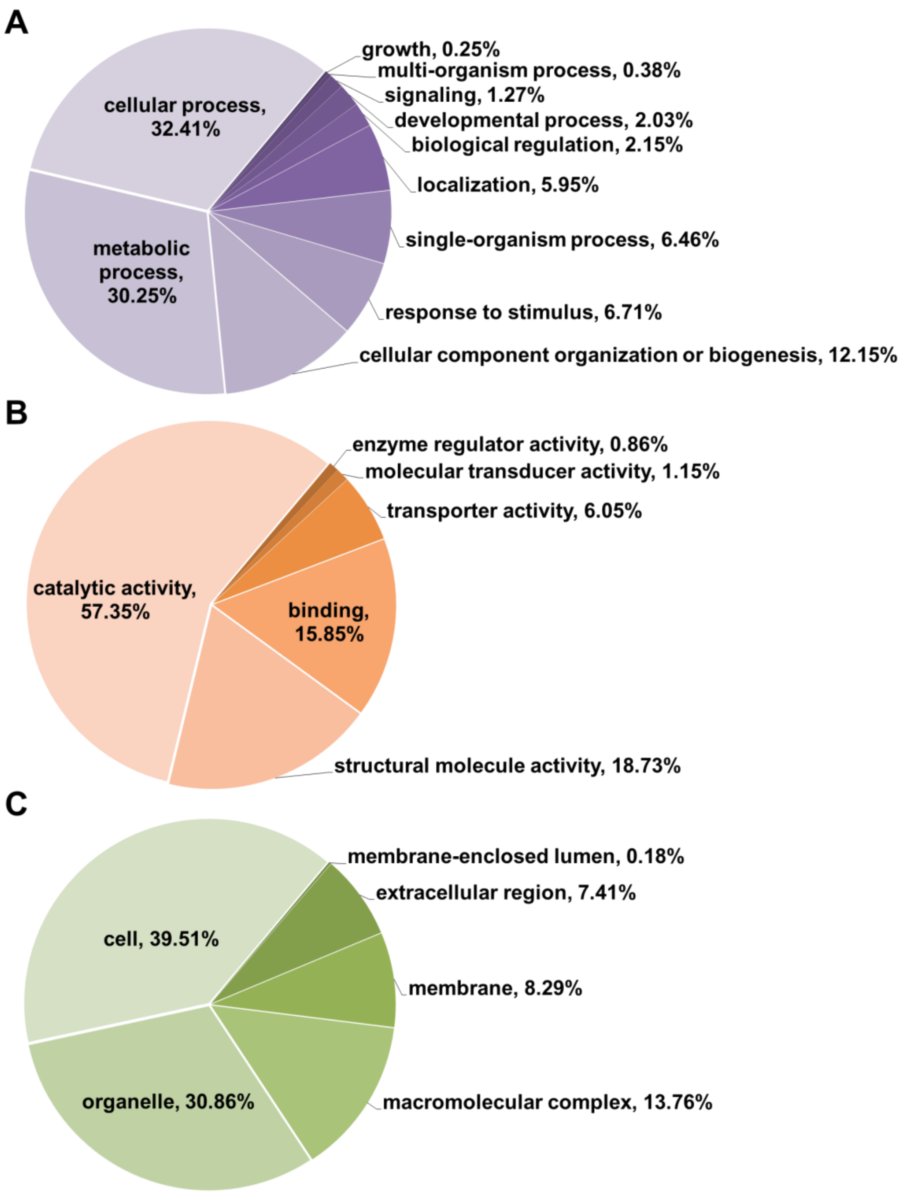

Supplementary Figure 5. Functional annotation of lysine succinylome according to biology processes (A), molecular functions (B) and celluar components (C).

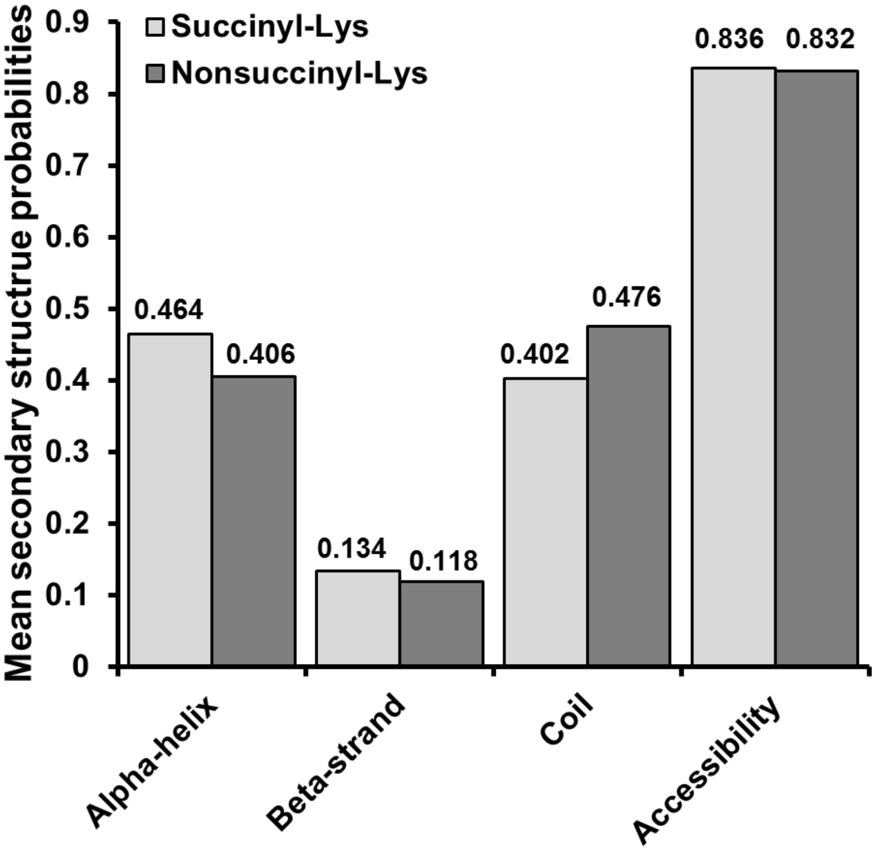

Supplementary Figure 6. Prediction of secondary structures and accessibility of succinylated and nonsuccinylated lysines.

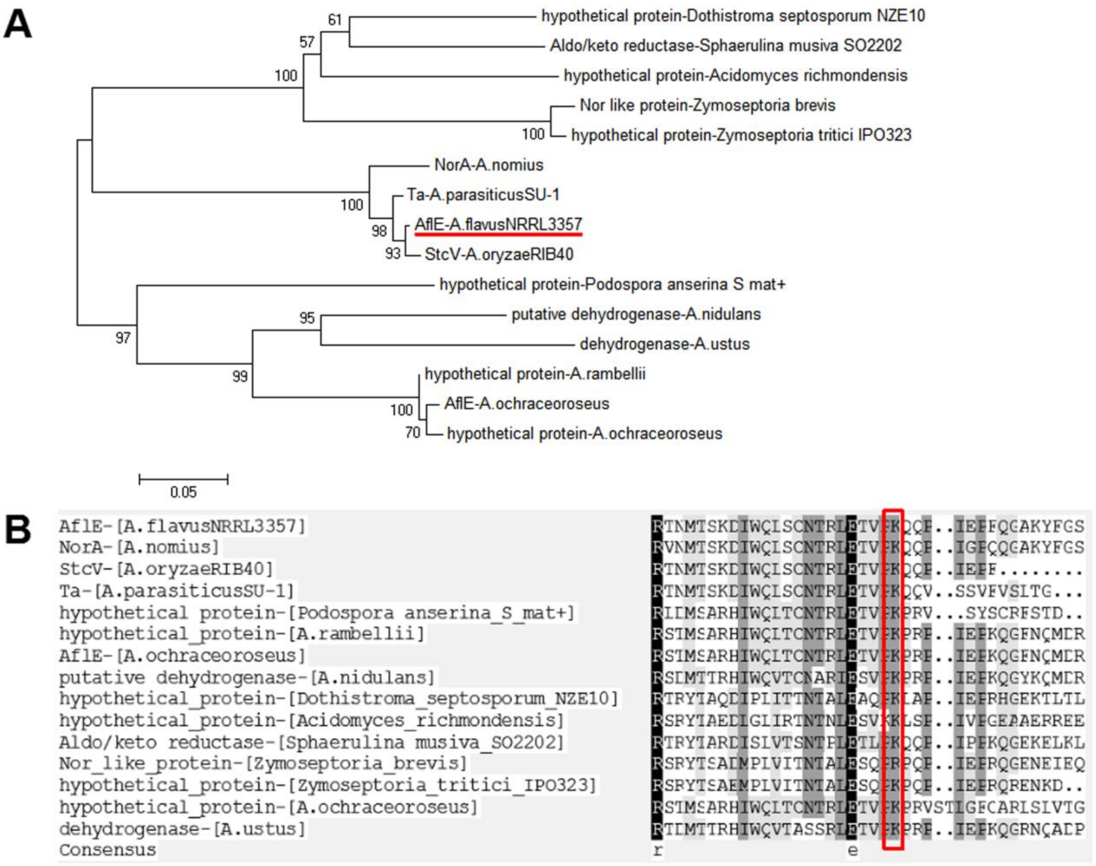

Supplementary Figure 7. The conservative analyses of AflE and its succinylated site. (A) Phylogenetic tree based on AflE protein in different species. (B) Multiple sequence alignment of AflE from different species. The conserved succinylation sites were marked by the red frame.

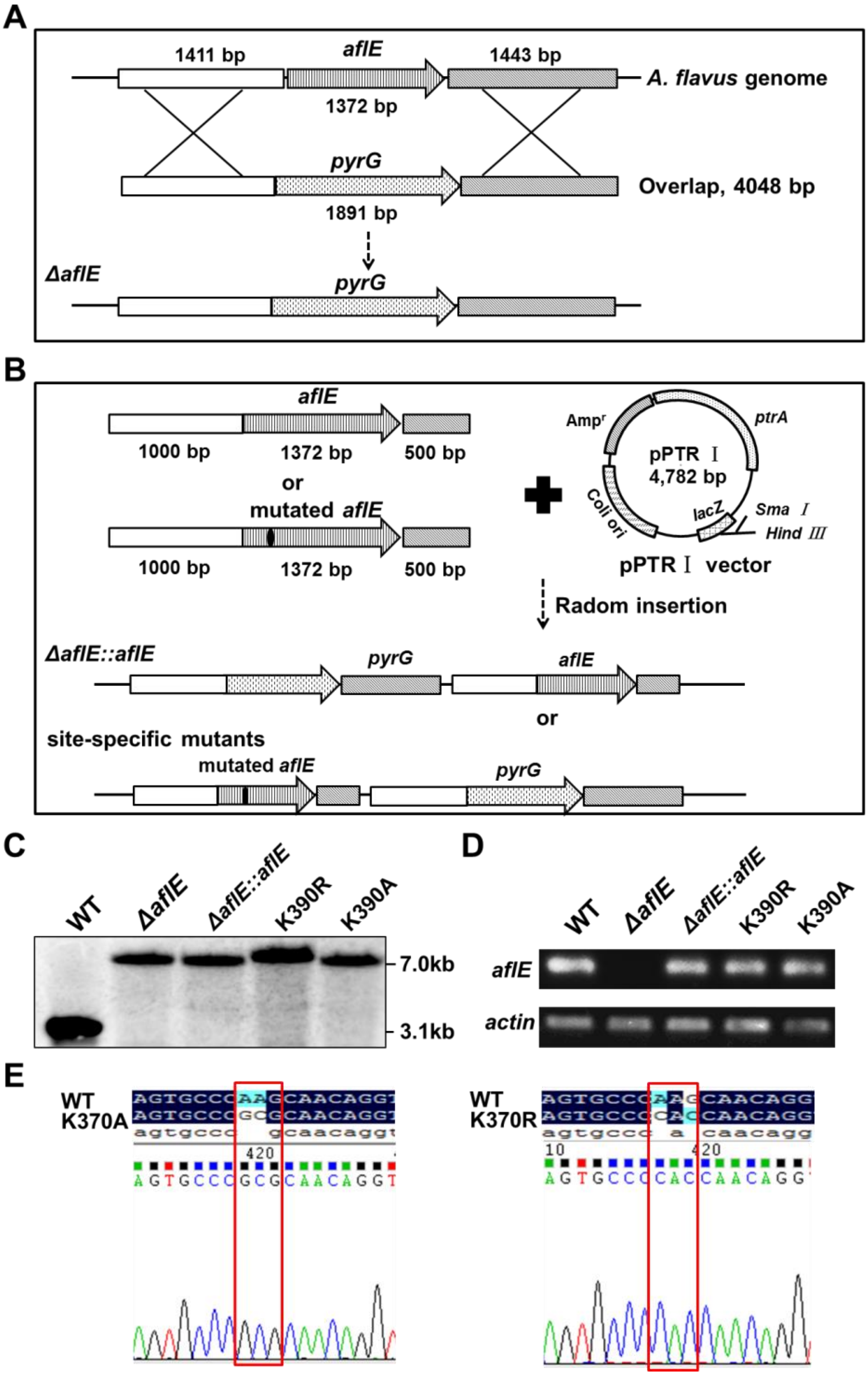

Supplementary Figure 8. Construction and confirmation of *aflE* mutants. (A) Schematic showing deletion of *aflE* gene in the genome of *A. flavus*. (B) Schematic showing the construction of complementary strain and site-specific mutants. (C) Southern blot analysis of the wild type, *ΔaflE*, and *ΔaflE*::*aflE* strains. DNA size for each hybridization band is shown on the right. (D) RT-PCR verification of *aflE* deletion. *actin* gene was used as a reference. (E) Verification of *aflE* mutations by DNA sequencing.

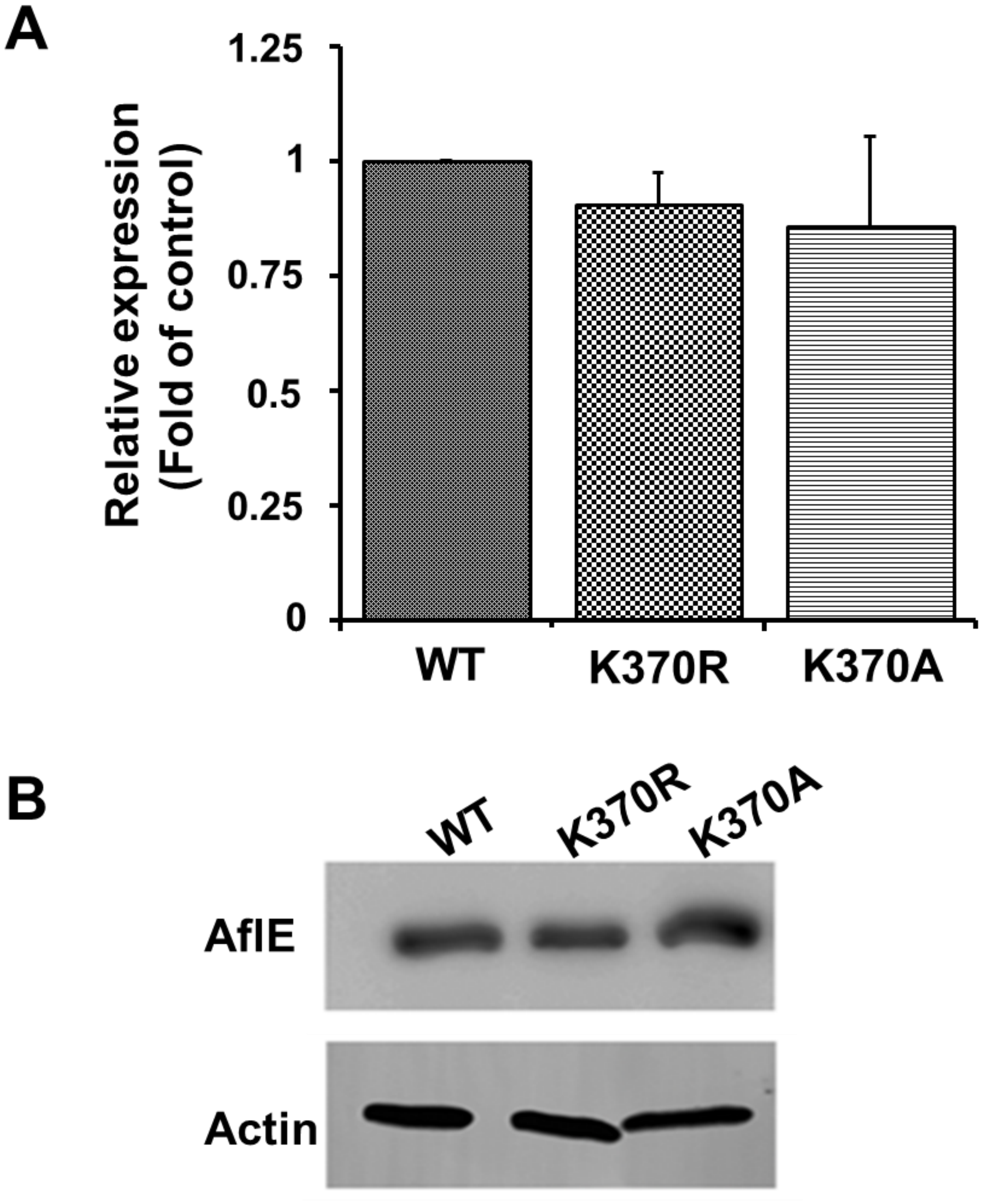

Supplementary Figure 9. Transcript level (A) and protein level (B) of *aflE* in the wild type, K370R and K370A strains.

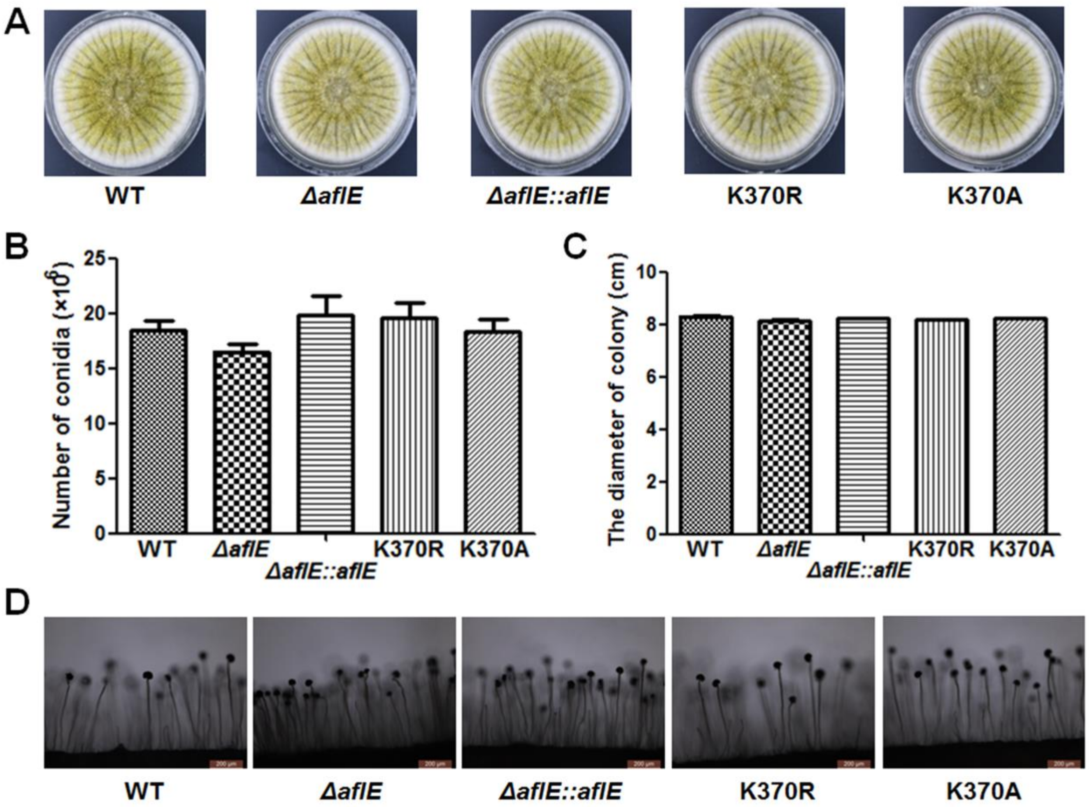

Supplementary Figure 10. Phenotypic analyses of different *A. flavus* strains. (A) Morphology analysis of colonies in WT, *ΔaflE*, K370R, K370A and Δ*aflE::aflE* strains on YES media. (B) Quantification analysis of conidia. Conidial production was counted from three replicates of PDA plates. (C) Measurement of colony diameter. Colony diameter was measured from three replicates of YES plates in (A). (D) Microscopic analysis of conidiophore structures (magnification: ×200). The WT, *ΔaflE*, K370R, K370A and Δ*aflE::aflE* strains were cultured on YES agar media.
