# Supplementary material for: Lysine Succinylation Contributes to Aflatoxin Production and Pathogenicity in Aspergillus flavus
Source: Mol Cell Proteomics. 2018 Jan 3;17(3):457–71. doi: 10.1074/mcp.RA117.000393 (PMC5836371; doi:10.1074/mcp.RA117.000393)
Supplement: Supplemental Data [file supp_RA117.000393_133046_1_supp_36568_p0skbz.doc]

**Supplemental Table 1. Primers used in this study.**

| **Gene name** | **Sequence** | **Application** |
| --- | --- | --- |
| *aflE*-1 | TCGCTGTCCGACTTAGAT | Amplified upstream of *aflE* |
| *aflE*-3 | GGGTGAAGAGCATTGTTTGAGGCGGGGCAGTAGGGAGAAC | Amplified upstream of *aflE* |
| *aflE*-4 | GCATCAGTGCCTCCTCTCAGACATTGTAGAACAGAGACACGCCT | Amplified downstream of *aflE* |
| *aflE*-6 | TTTCCACTTACCCATTCGG | Amplified downstream of *aflE* |
| *aflE*-2 | CTTCCGTTGACAGCAGAG | Amplified overlap of *aflE* |
| *aflE*-5 | CAGGTGACCGAACGATAC | Amplified overlap of *aflE* |
| *pyrG*-F | GCCTCAAACAATGCTCTTCACCC | Amplified *pyrG* |
| *pyrG*-R | GTCTGAGAGGAGGCACTGATGC | Amplified *pyrG* |
| *aflE*-C-F | CCCAAGCTTTCAGCGTGGAATACCG | Amplified complementation of *aflE* |
| *aflE*-C-R | TCCCCCGGGATGTTAGGCTGAAAG | Amplified complementation of *aflE* |
| *aflE*-O-F | ATGGCACAACGGCAGAAT | Amplified ORF of *aflE* |
| *aflE*-O-R | TTGAGGCAGAACCAAAGTA | Amplified ORF of *aflE* |
| *aflE*-K-A-F | CAGTGCCCGCGCAACAGGTGAGTAGTGTCTTTGTGTCC | Establishment K370A |
| *aflE*-K-A-R | GGACACAAAGACACTACTCACCTGTTGCGCGGGCACTG | Establishment K370A |
| *aflE*-K-R-F | CAGTGCCCCGGCAACAGGTGAGTAGTGTCTTTGTGTCC | Establishment K370R |
| *aflE*-K-R-R | GGACACAAAGACACTACTCACCTGTTGCCGGGGCACTG | Establishment K370R |
| *actin-*F | CAGCCGCTAAGAGTTCCAG | Amplified *actin* |
| *actin-R* | CACCGATCCAAACCGAGTAC | Amplified *actin* |
| *aflE*-QF | CGAGGAGGAAATCCGTGAAA | qPCR of *aflE* |
| *aflE*-QR | GCTCGTCATGTTAGTACGGTAG | qPCR of *aflE* |
| *aflR*-QF | AAAGCACCCTGTCTTCCCTAAC | qPCR of *aflR* |
| *actin*-QF | ACGGTGTCGTCACAAACTGG | qPCR of *actin* |
| *actin*-Q*R* | CGGTTGGACTTAGGGTTGATAG | qPCR of *actin* |
